# ﻿Discovery of the first resinicolous fungus in Mycosphaerellales (Dothideomycetes): *Resinomelaniacommunis* from conifer resins in Poland

**DOI:** 10.3897/mycokeys.120.154464

**Published:** 2025-07-29

**Authors:** Paweł Czachura, Marcin Piątek

**Affiliations:** 1 W. Szafer Institute of Botany, Polish Academy of Sciences, Lubicz 46, PL-31-512 Kraków, Poland W. Szafer Institute of Botany, Polish Academy of Sciences Kraków Poland

**Keywords:** Ascomycota, one new genus, one new species, Pinaceae, resinicolous fungi, taxonomy, Teratosphaeriaceae

## Abstract

Resinicolous fungi have evolved across different fungal lineages, but they are best known within the classes Eurotiomycetes, Leotiomycetes, Orbiliomycetes, and Xylonomycetes. Within the class Dothideomycetes, a resinicolous lifestyle is uncommon and is predominantly known in members of the order Mytilinidiales. In this study, 13 strains of a dark, slow-growing fungus belonging to Dothideomycetes were isolated from resins of four conifer species—*Abiesalba*, Larixdeciduassp.polonica, *Piceaabies*, and *Pinussylvestris* (all from the family Pinaceae)—in Poland. Based on multi-locus (LSU, ITS, *rpb2*, *tef1*, *tub2*) phylogenetic analyses and morphological features, the isolated strains were identified as representing an undescribed species in a new genus within the order Mycosphaerellales, family Teratosphaeriaceae. Consequently, a new genus and species, *Resinomelaniacommunis*, is described and illustrated. It is phylogenetically related to the genera *Neocatenulostroma* and *Sthughesia*. The common occurrence of *R.communis* on resins of four different conifer species indicates its resinicolous lifestyle. *Resinomelaniacommunis* is the first resinicolous fungus reported in Mycosphaerellales.

## ﻿Introduction

Resins play a wide protective role in plant defense, as they constitute both a physical and chemical barrier against harmful organisms. Due to their physical properties, resins coat wounds and engulf potential threats. In turn, their chemical composition protects plants from pests, herbivores, and pathogenic microorganisms (Langenheim 1994, 2003; Cabrita 2018). This chemical protection is made possible by their composition. In general, resins are a mixture of volatile and nonvolatile terpenoid and/or phenolic compounds. Gymnosperms (conifers) produce only terpenoid resins, whereas angiosperms produce both terpenoid and phenolic resins (Langenheim 1994, 2003; Cabrita 2018; Seyfullah et al. 2018). Such a composition makes resins recalcitrant to microbial decomposition and grants them antimicrobial properties (Langenheim 2003). Despite this, some fungi can live on such substrates. These are referred to as resinicolous fungi (Rikkinen and Poinar 2000; Tuovila et al. 2013; Beimforde et al. 2017a, b, 2020; Mitchell 2021).

Resinicolous fungi have evolved across different lineages within the fungal kingdom. They are best known within Eurotiomycetes (Chaetothyriales, Mycocaliciales), Leotiomycetes (Helotiales, Leotiales), Orbiliomycetes (Orbiliales), and Xylonomycetes (syn. Sareomycetes) (Xylonales, syn. Sareales). However, some species also occur in various scattered phylogenetic lineages (Rikkinen and Poinar 2000; Tuovila et al. 2013; Beimforde et al. 2017a,b, 2020; Baral et al. 2020; Hashimoto et al. 2021; Mitchell 2021; Mitchell et al. 2021; Czachura and Janik 2025). Resinicolous fungi are uncommon in the class Dothideomycetes. Within this class, only scattered reports of fungi exhibiting a resinicolous lifestyle exist, and their identification has mostly relied on morphological characters. Thus, their exact phylogenetic placement remains unknown (Mitchell 2021). The best-known examples—supported by DNA sequence data and phylogenetic analyses—are members of the order Mytilinidiales (Mitchell 2021; Czachura and Janik 2024). However, some reports based on morphologically identified species (Hughes 1968; Mitchell 2021), metabarcoding or recently published studies (Gøtzsche et al. 2024; Czachura et al. 2025) suggest that resinicolous fungi may be more common in Dothideomycetes than currently recognized.

During a study of fungi inhabiting resin of four conifer species in Poland (*Abiesalba*, Larixdeciduassp.polonica, *Piceaabies*, and *Pinussylvestris*; all from the family Pinaceae), 13 strains of a dark, slow-growing fungus belonging to the class Dothideomycetes were isolated. This was one of the most commonly isolated fungi from the resins of all studied conifer trees. Initial identification based on ITS rDNA sequences indicated an affinity with members of the order Mycosphaerellales. Based on multi-locus phylogenetic analyses and morphological features, the isolated strains were identified as representing an undescribed species in a new genus within the family Teratosphaeriaceae.

## ﻿Materials and methods

### ﻿Fungal isolation

Resin samples were collected from three different areas in central and southern Poland: the Modrzyna Reserve in the Beskid Niski Mountains, the Świętokrzyski National Park, and the Tatra National Park. Resin outpourings were sterilely collected from four conifer species—*Abiesalba*, Larixdeciduassp.polonica, *Piceaabies*, and *Pinussylvestris* (Pinaceae). In the laboratory, to remove external contamination, samples were washed with a 0.1% solution of Tween 20 and physiological saline. Details of the washing procedure are described in Czachura and Janik (2025). After drying, the outer resin layer was scraped using razor blades. Resin particles were spread on Petri dishes with four different media: dichloran–18% glycerol agar (DG18), dichloran rose bengal chloramphenicol agar (DRBC), rose bengal chloramphenicol agar (RBC), and potato dextrose agar (PDA). The mycological media were prepared as described by Crous et al. (2019) and Corry et al. (1995). Cultures containing resin particles were incubated in the dark at 15 °C or 25 °C. Selected representatives of each morphotype were transferred to malt extract agar (MEA) for storage prior to further processing. Thirteen strains of a dark, slow-growing fungus from the class Dothideomycetes were analyzed in detail in this study. All examined strains of *Resinomelaniacommunis* are preserved as dried cultures (KRAM F) and cultures stored under paraffin oil in the fungal collection of the W. Szafer Institute of Botany, Polish Academy of Sciences, Kraków, Poland. Representative cultures are also deposited in the culture collection (CBS) of the Westerdijk Fungal Biodiversity Institute, Utrecht, the Netherlands.

### ﻿Morphological analyses

Macroscopic features of the cultures were examined on 90 mm Petri dishes with four different media: malt extract agar (MEA), oatmeal agar (OA), potato dextrose agar (PDA), and synthetic nutrient-poor agar (SNA) after four weeks at 15 °C and 25 °C in darkness. Micromorphological features were studied on SNA. Micromorphology was observed on slides using a Nikon Eclipse 80i light microscope. Photographs were taken with the NIS‐Elements BR 3.0 imaging software. The figures showing morphological features of the analyzed strain were prepared using Inkscape 0.92.4.

### ﻿DNA extraction, amplification, and sequencing

Extraction of DNA was performed using the CTAB method as described in Czachura and Janik (2025). For phylogenetic analyses of isolated strains, five loci were amplified: the internal transcribed spacer 1 and 2 regions and intervening 5.8S rRNA gene (ITS), the partial 28S rRNA gene (LSU), the partial RNA polymerase II second largest subunit gene (*rpb2*), the partial translation elongation factor 1-α gene (*tef1*), and the partial β-tubulin gene (*tub2*). A polymerase chain reaction (PCR) mixture for each sample contained 17.25 μL cell culture grade water (Sigma Aldrich, USA), 2.5 μL of 10× PCR Buffer (Sigma Aldrich, USA), 2.5 μL of 25 mM MgCl_2_ (Sigma Aldrich, USA), 0.5 μL of 10 mM dNTPs (EURx, Poland), 0.5 μL of each 10 μM primer (Sigma Aldrich, USA), 0.25 μL of 5 U/μL Taq DNA Polymerase (Sigma Aldrich, USA), and 1 μL of isolated DNA. A fragment containing ITS and LSU was amplified using primers ITS1 and LR5 (White et al. 1990; Vilgalys and Hester 1990), whereas parts of protein-coding genes were amplified using primers fRPB2-5F and fRPB2-7cR for *rpb2* (Liu et al. 1999), EF1-728F and EF-2 for *tef1* (O’Donnell et al. 1998; Carbone and Kohn 1999), and T1 and β-Sandy-R for *tub2* (O’Donnell and Cigelnik 1997; Stukenbrock et al. 2012). The amplification of the fragment containing ITS and LSU was set as follows: an initial denaturation at 94 °C for 3 min, followed by 35 cycles of amplification (denaturation at 94 °C for 45 s, annealing at 50 °C for 45 s, and elongation at 72 °C for 2 min), and a final elongation step at 72 °C for 10 min. The amplification of *rpb2* was carried out under the following conditions: an initial denaturation at 94 °C for 3 min, followed by 35 cycles of amplification (denaturation at 94 °C for 60 s, annealing at 54 °C for 90 s, and elongation at 72 °C for 2 min), and a final elongation step at 72 °C for 10 min. Amplification conditions for *tef1* were conducted in the following steps: an initial denaturation at 94 °C for 3 min, followed by 40 cycles of amplification (denaturation at 94 °C for 45 s; annealing at 52 °C for 30 s; elongation at 72 °C for 90 s), and a final elongation step at 72 °C for 6 min. Amplification conditions for *tub2* were as follows: an initial denaturation at 94 °C for 3 min, followed by 40 cycles of amplification (denaturation at 94 °C for 45 s; annealing at 52 °C for 30 s; elongation at 72 °C for 90 s), and a final elongation step at 72 °C for 7 min. PCR products were visualized by agarose gel electrophoresis. Amplicons were enzymatically cleaned using an Exo-BAP Mix (EURx, Poland) according to the manufacturer’s instructions and submitted for bidirectional sequencing by Macrogen Europe B.V. (Amsterdam, The Netherlands). The ITS and LSU fragments were sequenced using primers ITS1 and ITS4 for ITS (White et al. 1990) and LSU1Fd and LR5 for LSU (Vilgalys and Hester 1990; Crous et al. 2009) to ensure high-quality sequences across the full amplicon lengths. Protein-coding loci were sequenced using the same primer pairs as those used for amplification.

### ﻿Phylogenetic analyses

Sequence assembly and preparation of sequence alignments were carried out using Geneious Prime 2022.1.1. Newly generated forward and reverse sequences were assembled and trimmed. To determine the closest taxonomic affinities of the obtained sequences, a megablast search was performed in the NCBI GenBank nucleotide database (https://www.ncbi.nlm.nih.gov/genbank/) (Zhang et al. 2000). Phylogenetic analyses were conducted using combined LSU, ITS, *rpb2*, *tef1*, and *tub2* sequences of representatives of the Teratosphaeriaceae. Sequences used in the phylogenetic analyses are listed in Table 1. Reference sequences were derived from ex-type strains (whenever possible) of the type species of all genera assigned to Teratosphaeriaceae (Hyde et al. 2024). Additionally, sequences of all *Xenomeris* strains available in GenBank were included in the analyses, as *Xenomerisjuniperi* (current name: *Sthughesiajuniperi*) was identified as one of the closest relatives of the analyzed strains in the megablast search. Each single-locus dataset was aligned separately using the MAFFT algorithm (Katoh et al. 2019). Maximum likelihood (ML) and Bayesian inference (BI) analyses were used to estimate phylogenetic relationships. ML analysis was conducted using RAxML-NG v. 1.2.2 (Kozlov et al. 2019), with 1,000 bootstrap replicates, and BI analysis was performed using MrBayes v. 3.2.6 (Ronquist et al. 2012). The most suitable substitution models for each locus were selected separately using ModelTest-NG v. 0.2.0 (Darriba et al. 2020), based on the Bayesian Information Criterion (BIC). Substitution models applied in ML and BI analyses are provided in Table 2. For the BI analysis, four simultaneous Markov Chain Monte Carlo (MCMC) chains were run for 50,000,000 generations with a sampling frequency of 100 generations. The first 25% of sampled trees were discarded as burn-in, and the remaining trees were used to reconstruct the consensus tree and calculate posterior probability values for branches. The ML and BI phylogenetic trees were visualized using FigTree v. 1.4.3. The final tree topology was based on the ML analysis, with maximum likelihood bootstrap (MLB) and Bayesian posterior probability (BPP) values indicated next to branches. The final phylogenetic tree was graphically edited in Inkscape 0.92.4.

**Table 1.** GenBank accession numbers and details of strains used in the phylogenetic analyses.

Species	Strain	Isolation source	Location	GenBank accession numbers				
				LSU	ITS	*rpb2*	*tef1*	*tub2*
*Acidiellabohemica*	CBS 132721^T^	Highly acidic soil	Czech Republic	KF901985	–	KF902179	–	–
*Acidomycesacidophilus*	CBS 335.97	Acidophilic algae *Dunaliellaacidophila*, pH 1.0	Germany	MH874256	AJ244237	–	–	–
*Acrodontiumcrateriforme*	CBS 144.33^T^	–	Netherlands	KX286952	FN666565	–	–	–
*Apenidiellastrumelloidea*	CBS 114484^T^	*Carex* sp.	Russia	KF937229	EU019277	KF937266	–	–
*Araucasphaeriafoliorum*	CPC 33084	*Araucariaaraucana*	Chile	MH327829	MH327793	–	–	–
*Aulographinapinorum*	CBS 174.90	*Pinusinsignis*, needles	France	GU301802	–	GU371737	–	–
*Austroafricanaassociata*	CBS 120730^T^ = CPC 13119	*Corymbiahenryii*	Australia	KF901824	KF901507	KF902177	KF903087	KF902790
*Austrostigmidiummastodiae*	MA 18215^T^	*Mastodiatessellata*	Antarctica	KP282859	–	–	–	–
*Batcheloromycesproteae*	CBS 110696^ET^	*Proteacynaroides*	South Africa	KF901833	JF746163	KF902195	–	–
*Baudoiniacompniacensis*	UAMH 10808^ET^	Tree branches near a distillery	France	KT186489	KT186468	–	KT186533	KT186511
*Bryochitonmonascus*	CBS 126284	Bryophyte	Finland	MH875416	MH863957	–	–	–
*Caatingomycesbrasiliensis*	URM 7916^T^	*Poincianellapyramidalis*	Brazil	MH929439	MH929437	–	MH929443	–
*Caeliomycestampanus*	CBS 148275^T^ = CPC 41060	Office dust	USA	OK663765	OK664726	–	OK651189	OK651207
*Camarosporulapersooniae*	CBS 112494 = CPC 3350	*Persoonia* sp.	Australia	JF770460	JF770448	KF937255	–	–
*Capnobotryellarenispora*	CBS 214.90	*Capnobotrysneessi*	Japan	GU214398	AY220612	–	–	–
*Catenulostromaprotearum*	CBS 125421^ET^ = CPC 15370	*Leucadendrontinctum*	South Africa	KF902090	MH863677	KF902198	–	–
*Constantinomycesvirgultus*	CBS 117930^T^ = TRN79	Rock, limestone	Spain	GU323964	AY559339	KF310082	–	KF546781
*Devriesiastaurophora*	DAOM 60786^T^	*Pinussylvestris*, decaying needle	United Kingdom	–	AY692085	–	–	–
*Elasticomyceselasticus*	CBS 122538^T^ = CCFEE 5313	Lichen thallus (*Usneaantarctica*)	Antarctica	KJ380894	FJ415474	–	–	–
*Eupenidiellavenezuelensis*	CBS 106.75^T^	*Homosapiens*, tinea nigra	Venezuela	KF902163	KF901802	KF902202	KF903100	KF902803
*Euteratosphaeriaverrucosiafricana*	CBS 118496^T^ = CPC 11167	*Eucalyptus* sp.	Indonesia	–	DQ303056	–	–	–
*Friedmanniomycesendolithicus*	CCFEE 5208^T^	Rock	Antarctica	–	JN885549	–	–	–
*Haniomycesdodonaeae*	KUMCC 20-0220^T^	*Dodonaeaviscosa*, dead twigs	China	MW264191	MW264212	MW269527	MW256813	–
*Hispidoconidiomaalpinum*	UAMH 11010^T^	*Larixlyallii*	Canada	FJ997286	FJ997285	–	–	–
*Hortaeawerneckii*	CBS 107.67^T^	*Homosapiens*, tinea nigra	Portugal	EU019270	AJ238468	–	MH259537	–
*Hyweljonesiaqueenslandica*	BRIP 61322b^T^	Cocoon of an unidentified microlepidoptera	Australia	KY173562	KY173476	–	–	–
*Incertomycesperditus*	CBS 136105^T^ = CCFEE 5385	Rock	Italy	KF310008	KF309977	KF310083	–	KF546733
*Intumescentiatinctorum*	CGMCC 3.23634^T^	*Parmotrematinctorum*	China	OP345116	OP345149	OP354472	OP354466	–
*Lapidomyceshispanicus*	CBS 118355^T^ = TRN500	Rock	Spain	KF310017	AY843182	–	–	KF546778
*Lawreyaglyphidiphila*	C9506N	*Glyphisscyphulifera*	USA	MK693147	–	–	–	–
*Meristemomycesfrigidus*	CBS 136109^T^ = CCFEE 5508	Rock	Argentina	GU250401	KF309971	KF310067	–	KF546750
*Microcyclosporapomicola*	CBS 126141^T^ = CPC 16175	*Malusdomestica*, fruit surface	Germany	MH875506	MH864049	–	–	–
*Monticolaelongata*	CBS 136206^T^ = CCFEE 5394	Rock	Italy	KF309995	KF309965	KF310062	–	KF546735
*Muriphilaoklahomaensis*	CBS 146146^T^ = CCF 5751	Outside wall of alcohol distillery	USA	LR736041	LR736040	–	–	–
*Myrtapenidiellatenuiramis*	CBS 124993^T^ = CPC 13692	*Eucalyptustenuiramis*	Australia	GQ852626	KF901658	KF937262	KF903121	KF442484
*Neobryochitonnarthecii*	CBS 149172^T^ = CPC 41972	*Nartheciumossifragum*, dead leaves	Netherlands	ON811567	ON811508	–	–	–
*Neocatenulostromaabietis*	CBS 459.93^T^	*Abies* sp.	Germany	NG_057756	NR_145103	–	–	–
*Neocatenulostromacastaneae*	MFLUCC 17–2188^T^	*Castaneasativa*, dead branches	Italy	MZ518791	MZ519072	–	–	–
*Neocatenulostromagermanicum*	CBS 539.88^T^	Rock	Germany	MH873835	MH862143	KF902231	–	–
*Neocatenulostromamicrosporum*	CBS 101951^T^ = CPC 1960	*Proteacynaroides*, living leaf	South Africa	KF901814	KF901499	KF902232	KF903122	KF902825
*Neocatenulostromaspinulosum*	CBS 150899^T^ = FMR 18793	PVC pipe	Spain	OX628945	OX628944	–	–	–
*Neophaeothecoideaproteae*	CBS 114129^T^ = CPC 2831	*Protearepens*	South Africa	KF937228	EU707898	KF937265	–	–
*Neotrimmatostromaexcentricum*	CBS 121102^T^ = CPC 13092	*Eucalyptusagglomerata*	Australia	KF901840	KF901518	KF902236	KF903123	KF902826
*Nothodevriesianarthecii*	CBS 149066^T^ = CPC 42166	*Nartheciumossifragum*, dead leaves	Netherlads	ON603799	ON603779	–	ON605631	ON605639
*Oleoguttulamirabilis*	CBS 136102^T^ = CCFEE 5523	Rock	Antarctica	KF310031	KF309973	–	–	–
*Palmeiromyceschamaeropicola*	CDP 001^T^	*Chamaeropshumilis*, leaf spots	Portugal	MT076194	MT068628	–	–	–
*Parapenidiellatasmaniensis*	CBS 111687^T^ = CMW 14780 = CPC 1555	*Eucalyptusnitens*	Australia	KF901843	KF901521	KF902263	KF903150	KF902853
*Parateratosphaeriabellula*	CBS 111700^ET^ = CPC 1821	*Proteaeximia*	South Africa	KF937232	MH862879	–	–	–
*Penidiellacolumbiana*	CBS 486.80^T^	*Paepalanthuscolumbianus*	Colombia	KF901965	KF901630	KF902272	KF903158	KF902860
*Penidiellomycesaggregatus*	CBS 128772^T^ = CPC 18278	*Phaenocomaprolifera*, leaf bracts	South Africa	JF499862	JF499842	–	–	–
*Penidiellopsisradicularis*	CBS 140695^T^ = UTHSC DI-13-256	*Homosapiens*, nail	USA	LN834445	LN834441	–	–	–
*Phaeothecoidea eucalypti*	CBS 120831^T^ = CPC 12918	*Eucalyptusbotryoides*	Australia	KF901848	KF901526	KF902285	KF903170	KF902872
*Pseudoramichloridiumhenryi*	CBS 124775^T^ = CPC 13121	*Corymbiahenryi*	Australia	KF901857	KF901535	KF902344	KF903227	KF902930
*Pseudotaeniolinaglobosa*	CBS 109889^T^	Rock, sandstone	Italy	EU019283	AY128700	MW371115	–	MW371112
*Pseudoteratosphaeriaperpendicularis*	CBS 118367^T^ = CPC 10983	*Eucalyptuseurograndis*	Colombia	KF901972	KF901637	KF902350	KF903232	KF902936
*Queenslandipenidiellakurandae*	CBS 121715^T^ = CPC 13333	Unidentified tree, exudates of bleeding stem cankers	Australia	KF901860	KF901538	KF902356	KF903238	KF902942
*Rapidomycesnarthecii*	CBS 149174^T^ = CPC 41974	*Nartheciumossifragum*, dead leaves	Netherlands	ON811589	ON811532	–	–	–
*Readeriellamirabilis*	CBS 125000^ET^ = CPC 12370	*Eucalyptusglobulus*	Australia	KF901871	KF901549	KF902376	KF903257	KF902960
*Recurvomycesmirabilis*	CBS 119434^T^ = CCFEE 5264	Rock, sandstone	Antarctica	GU250372	FJ415477	KF310059	–	–
***Resinomelaniacommunis***	**CBS 152715^T^ = P0008**	**Resin of *Piceaabies***	**Poland**	**PV166125**	**PV166112**	**PV172631**	**PV172638**	**PV172651**
***Resinomelaniacommunis***	**CBS 152716 = P0010**	**Resin of *Piceaabies***	**Poland**	**PV166126**	**PV166113**	**PV172632**	**PV172639**	**PV172652**
***Resinomelaniacommunis***	**CBS 152717 = P0012**	**Resin of *Pinussylvestris***	**Poland**	**PV166127**	**PV166114**	**PV172633**	**PV172640**	**PV172653**
***Resinomelaniacommunis***	**CBS 152718 = P0014**	**Resin of *Pinussylvestris***	**Poland**	**PV166128**	**PV166115**	**PV172634**	**PV172641**	**PV172654**
***Resinomelaniacommunis***	**CBS 152719 = P0017**	**Resin of *Abiesalba***	**Poland**	**PV166129**	**PV166116**	**PV172635**	**PV172642**	**PV172655**
***Resinomelaniacommunis***	**CBS 152720 = P0019**	**Resin of Larixdeciduassp.polonica**	**Poland**	**PV166130**	**PV166117**	**PV172636**	**PV172643**	**PV172656**
***Resinomelaniacommunis***	**P0009**	**Resin of *Piceaabies***	**Poland**	**PV166131**	**PV166118**	**PV172637**	**PV172644**	**PV172657**
***Resinomelaniacommunis***	**P0011**	**Resin of *Piceaabies***	**Poland**	**PV166132**	**PV166119**	–	**PV172645**	**PV172658**
***Resinomelaniacommunis***	**P0015**	**Resin of *Pinussylvestris***	**Poland**	**PV166133**	**PV166120**	–	**PV172646**	**PV172659**
***Resinomelaniacommunis***	**P0016**	**Resin of *Abiesalba***	**Poland**	**PV166134**	**PV166121**	–	**PV172647**	**PV172660**
***Resinomelaniacommunis***	**P0018**	**Resin of *Abiesalba***	**Poland**	**PV166135**	**PV166122**	–	**PV172648**	**PV172661**
***Resinomelaniacommunis***	**P0020**	**Resin of Larixdeciduassp.polonica**	**Poland**	**PV166136**	**PV166123**	–	**PV172649**	**PV172662**
***Resinomelaniacommunis***	**P0021**	**Resin of Larixdeciduassp.polonica**	**Poland**	**PV166137**	**PV166124**	–	**PV172650**	**PV172663**
*Salinomycespolonicus*	CBS 147480^T^	Brine	Poland	MW581538	MW581542	MW583643	–	–
*Simplicidiellanigra*	AP 416^T^ = CBMAI 1939	*Attacapiguara*	Brazil	KU216313	KT833147	–	KU216338	KU216266
*Staninwardiasuttonii*	CBS 120061	*Eucalyptusrobusta*	Australia	KF901874	KF901552	KF902392	KF903270	KF902974
*Stenellaaraguata*	CBS 105.75^T^ = ATCC 24788 = FMC 245	*Homosapiens*, tinea nigra	Venezuela	MH872633	EU019250	–	–	–
*Sthughesiajuniperi*	xejucf	*Juniperusoccidentalis*	USA	EF114709	EF114689	–	–	–
*Suberoteratosphaeriasuberosa*	CBS 436.92^T^ = CPC 515	*Eucalyptusdunnii*	Brazil	KF901949	KF901623	KF902404	KF903282	–
*Teratoramulariapersicariae*	CBS 141105^T^ = CPC 11410	*Persicarianepalensis*	South Korea	KX287252	KX287548	KX288713	KX288110	–
*Teratosphaeriafibrillosa*	CBS 121707^ET^ = CPC 13960	*Protea* sp.	South Africa	KF902075	KF901728	–	KF903305	KF903003
*Teratosphaericolapseudoafricana*	CBS 114782^T^ = CPC 1230	*Eucalyptusglobulus*	Zambia	KF902084	KF901737	KF902500	KF903371	KF903067
*Teratosphaeriopsispseudoafricana*	CBS 111171^T^ = CPC 1261	*Eucalyptus* sp.	South Africa	KF902085	KF901738	KF902501	KF903372	KF903068
*Xanthoriicolaphysciae*	CR22	*Xanthoriaparietina*	United Kingdom	JN040491	–	–	–	–
*Xenoconiothyriumcatenatum*	CBS 128994^T^ = CMW 22113	Twig litter of *Protealaurifolia*	South Africa	JN712570	JN712502	–	–	–
*Xenomerisraetica*	CBS 485.61	*Arctostaphylosuva*-*ursi*	Switzerland	EF114716	EF114690	–	–	–
*Xenomeris* sp.	AM224-P6T3N	Pinyon tree, needle	USA	–	KT264511	–	–	–
*Xenomeris* sp.	AM519-P4T11N	Pinyon tree, needle	USA	–	KT264669	–	–	–
*Xenopenidiellarigidophora*	CBS 314.95^T^	Leaf litter of *Smilax* sp.	Cuba	MH874156	EU019276	KF902507	–	–
*Xenophacidiellapseudocatenata*	CBS 128776^T^ = CPC 18472	*Phaenocomaprolifera*	South Africa	JF499870	JF499850	KF902508	–	–
*Xenoteratosphaeriajonkershoekensis*	CBS 122897^ET^ = CPC 13984	*Protea* sp.	South Africa	KF937250	EU707864	KF937285	–	–

Sequences and details of strains obtained in this study are shown in bold. Abbreviations: T: ex-holotype; ET: ex-epitype; –: indicates unavailable data or sequence.

**Table 2.** Substitution models used in the phylogenetic analyses.

Partitions	ML	MB
LSU	TrN+I+G4	GTR+I+G4
ITS	TIM1+I+G4	SYM+I+G4
*rpb2* ^1st^	TIM3ef+I+G4	SYM+I+G4
*rpb2* ^2nd^	TPM2uf+G4	HKY+G4
*rpb2* ^3rd^	TPM2+I+G4	SYM+I+G4
*tef1* ^intron^	HKY+I+G4	HKY+I+G4
*tef1* ^1st^	TrNef+I+G4	JC+I+G4
*tef1* ^2nd^	JC+I	JC+I
*tef1* ^3rd^	TrN+G4	HKY+G4
*tub2* ^1st^	TPM3uf+G4	HKY+G4
*tub2* ^2nd^	JC+I	JC+I
*tub2* ^3rd^	TPM1uf+G4	HKY+G4
*tub2* ^intron1^	TPM3uf+G4	K80+G4
*tub2* ^intron2^	HKY+I+G4	HKY+I+G4

ML = maximum likelihood phylogenetic inference; MB = Bayesian phylogenetic inference; superscripts ^1st^, ^2nd^, ^3rd^ represents the first, second and third codon positions of the exon, respectively.

## ﻿Results

### ﻿Phylogenetic analyses

**Figure 1.**
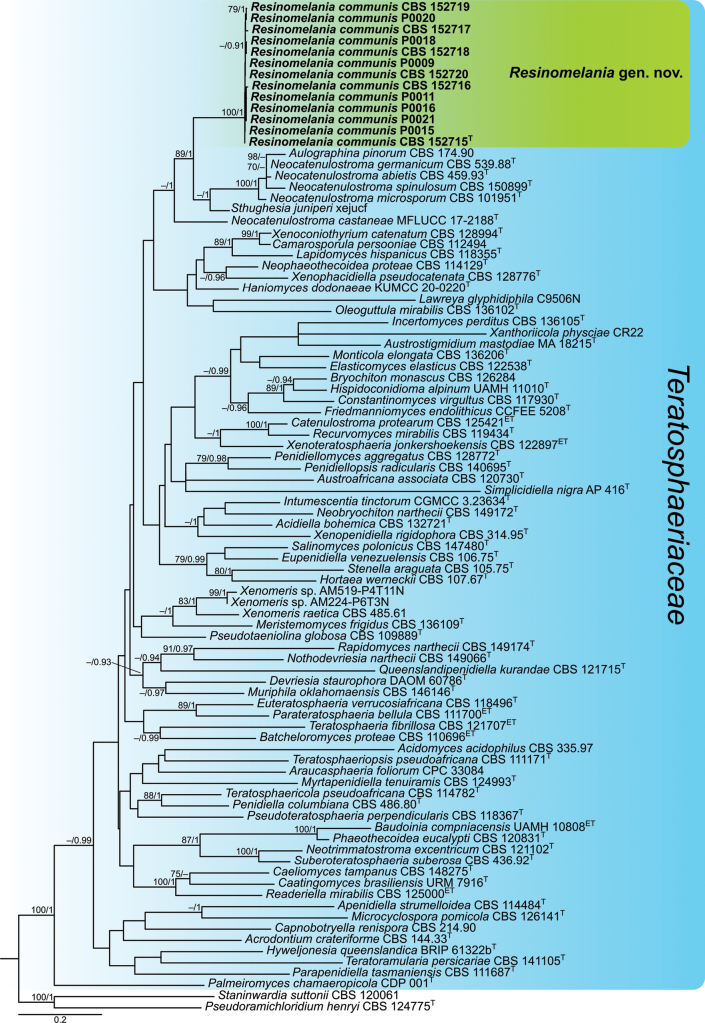
Maximum likelihood consensus tree inferred from the combined LSU, ITS, *rpb2*, *tef1*, and *tub2* multiple sequence alignment of representatives of the family Teratosphaeriaceae. The position of *Resinomelaniacommunis* is indicated in bold and marked by a colored block. Maximum likelihood bootstrap (MLB) support values ≥70% and Bayesian posterior probabilities (BPP) ≥0.9 are shown next to the branches (MLB/BPP). Ex-type and ex-epitype strains are indicated with T and ET, respectively. The scale bar represents the average number of substitutions per site.

The phylogenetic placement of the analyzed strains was determined using multi-locus phylogenetic analyses based on the combined LSU, ITS, *rpb2*, *tef1*, and *tub2* dataset. ML and BI analyses yielded similar tree topologies. The tree obtained from the ML analysis is presented in Fig. 1. Maximum likelihood bootstrap (MLB) support values above 70% and Bayesian posterior probabilities (BPP) above 0.9 were considered well supported, and only those values are shown next to the branches.

The phylogenetic analyses inferred that all genera in the family Teratosphaeriaceae formed a well-supported clade (MLB/BPP = 100/1), although most internal branches were weakly supported. As a result, the relationships among genera within Teratosphaeriaceae were largely unresolved, with some exceptions, including the group analyzed in this study. Sequences of *Xenomeris* species formed two distinct lineages: one composed of *X.raetica* and *Xenomeris* sp., which was related to *Meristemomycesfrigidus* (MLB/BPP = 45/1); and another containing *Xenomerisjuniperi*, which was sister to *Neocatenulostroma* (MLB/BPP = 63/1). The latter species is also known under the combination *Sthughesiajuniperi*, the type species of the genus *Sthughesia*. Sequences of the analyzed strains from resin samples formed a fully supported, independent lineage (MLB/BPP = 100/1) that clustered as a sister group to most members of the genera *Neocatenulostroma* and *Sthughesia*, both of which also formed independent lineages. The only exception was *Neocatenulostromacastaneae*, which formed a distinct single-species lineage distant from other *Neocatenulostroma* species, including the type species, *N.microsporum*. The phylogenetic distance from *Neocatenulostroma* and *Sthughesia* species suggests that the strains analyzed from conifer resins represent an undescribed species belonging to a novel genus within the family Teratosphaeriaceae.

### ﻿Taxonomy

#### ﻿ Resinomelania

Taxon classificationFungiMycosphaerellalesTeratosphaeriaceae

Czachura & Piątek gen. nov.

A6BB7CF4-811D-5FB4-9F14-662D4841D21A

859781

##### Etymology.

The genus name refers to a resinicolous lifestyle and the dark, melanized mycelium of this fungus.

##### Description.

Mycelium composed of branched, septate, hyaline, subhyaline, pale brown to brown, smooth or verruculose, cylindrical and moniliform hyphae, with hyphal swellings. Chlamydospores subglobose or broadly ellipsoidal, pale brown to brown, smooth or slightly verruculose, aseptate or muriformly septate, intercalary or terminal, single. Arthroconidia broadly ellipsoidal, one-septate or rarely aseptate, mostly constricted at septa, pale brown to brown, smooth or verruculose, intercalary or terminal, in chains.

##### Type species.

*Resinomelaniacommunis* Czachura & Piątek.

#### ﻿ Resinomelania communis

Taxon classificationFungiMycosphaerellalesTeratosphaeriaceae

Czachura & Piątek sp. nov.

7331A166-5292-5BE3-98E5-2000BB47AA74

859782

Figs 2 , 3

##### Etymology.

The epithet is derived from the Latin word that means ‘‘common’’ and refers to the common occurrence of this species on conifer resins.

**Figure 2.**
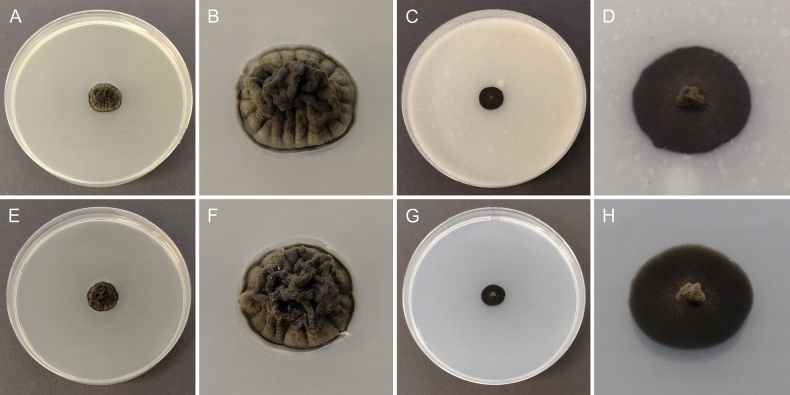
Morphology of cultures of *Resinomelaniacommunis* (CBS 152715) after 4 weeks at 25°C: **A, B.** Colony on MEA; **C, D.** Colony on OA; **E, F.** Colony on PDA; **G, H.** Colony on SNA.

##### Typus.

Poland • Świętokrzyskie Province, Kielce County, the Świętokrzyski National Park, Mt. Łysica, on resin of *Piceaabies*, 18 June 2021, leg. P. Czachura (holotype: KRAM F-60010; culture ex-type: CBS 152715 = P0008).

**Figure 3.**
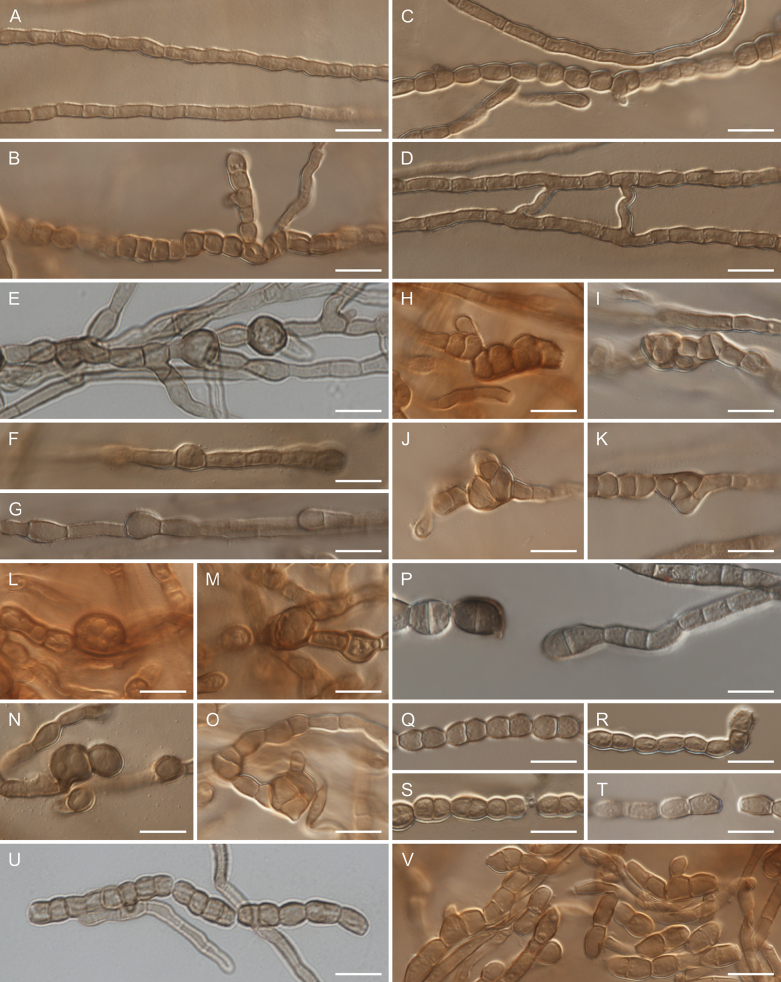
Morphology of *Resinomelaniacommunis* (CBS 152715): **A.** Cylindrical hyphae; **B.** Moniliform hyphae; **C.** Cylindrical and moniliform hyphae; **D.** Anastomosing hyphae; **E–G.** Aseptate hyphal swellings; **H–K.** Hyphal swellings in form of multicellular bodies; **L–O.** Chlamydospores; **P–V.** Arthroconidia. Scale bars: 10 μm.

##### Description.

Mycelium composed of branched, septate, hyaline to subhyaline (when young) or pale brown to brown (when mature), smooth or verruculose, guttulate, sometimes anastomosing, cylindrical (2.0–5.0 μm wide) and moniliform hyphae (3.5–9.5 μm wide). Hyphae with numerous aseptate, one-septate, or multiseptate hyphal swellings, 5.0–9.5 μm wide; multiseptate hyphal swellings in form of multicellular bodies, which are formed by meristematic development of hyphae. Chlamydospores subglobose or broadly ellipsoidal, pale brown to brown, smooth or slightly verruculose, aseptate or muriformly septate, 6.5–12.5 × 6.0–10.0 μm, intercalary or terminal, single, guttulate. Arthroconidia formed by arthric disarticulation, broadly ellipsoidal, one-septate or rarely aseptate, mostly constricted at septa, pale brown to brown, smooth or verruculose, 6.0–12.5 × 4.5–7.0 μm, intercalary or terminal, in chains, guttulate.

##### Culture characteristics.

Colonies on MEA and PDA convex, with folded surface, grey olivaceous to smoke grey, margin slightly lobate to slightly crenate; on MEA reaching 14 mm diam. after 4 weeks at 15 °C and 16 mm diam. after 4 weeks at 25 °C, reverse grayish black; on PDA reaching 12 mm diam. after 4 weeks at 15 °C and 16 mm diam. after 4 weeks at 25 °C, reverse grayish black. Colonies on OA and SNA flat with protrusion at center, fuscous black, margin nearly entire to slightly undulate; on OA reaching 10 mm diam. after 4 weeks at 15 °C and 12 mm diam. after 4 weeks at 25 °C, reverse grayish black; on SNA reaching 6 mm diam. after 4 weeks at 15 °C and 12 mm diam. after 4 weeks at 25 °C, reverse grayish black.

##### Additional specimens examined.

Poland • Małopolskie Province, Tatra County, the Tatra National Park, Dolina Strążyska, on resin of *Piceaabies*, 23 Sept. 2020, leg. P. Czachura & M. Piątek (KRAM F-60012; culture: CBS 152716 = P0010); • Podkarpackie Province, Krosno County, the Modrzyna Reserve, on resin of *Pinussylvestris*, 22 Oct. 2020, leg. P. Czachura (KRAM F-60014; culture: CBS 152717 = P0012); • Świętokrzyskie Province, Kielce County, the Świętokrzyski National Park, the strict protection area Mokry Bór, on resin of *Pinussylvestris*, 18 June 2021, leg. P. Czachura (KRAM F-60016; culture: CBS 152718 = P0014); • Świętokrzyskie Province, Kielce County, the Świętokrzyski National Park, Psarska Góra, on resin of *Abiesalba*, 15 Oct. 2020, leg. P. Czachura (KRAM F-60019; culture: CBS 152719 = P0017); • Podkarpackie Province, Krosno County, the Modrzyna Reserve, on resin of Larixdeciduassp.polonica, 22 June 2021, leg. P. Czachura (KRAM F-60021; culture: CBS 152720 = P0019); • Świętokrzyskie Province, Kielce County, the Świętokrzyski National Park, the strict protection area Psarski Dół, on resin of *Piceaabies*, 16 Oct. 2020, leg. P. Czachura (KRAM F-60011; culture: P0009); • Małopolskie Province, Tatra County, the Tatra National Park, Dolina Filipka, on resin of *Piceaabies*, 15 July 2021, leg. P. Czachura & M. Piątek (KRAM F-60013; culture: P0011); • Świętokrzyskie Province, Kielce County, the Świętokrzyski National Park, Dolina Czarnej Wody, on resin of *Pinussylvestris*, 15 Oct. 2020, leg. P. Czachura (KRAM F-60017; culture: P0015); • Podkarpackie Province, Krosno County, the Modrzyna Reserve, on resin of *Abiesalba*, 21 June 2021, leg. P. Czachura (KRAM F-60018; culture: P0016); • Małopolskie Province, Tatra County, the Tatra National Park, Łysa Skałka, on resin of *Abiesalba*, 08 Oct. 2020, leg. P. Czachura & M. Piątek (KRAM F-60020; culture: P0018); • Świętokrzyskie Province, Kielce County, the Świętokrzyski National Park, Chełmowa Góra, on resin of Larixdeciduassp.polonica, 16 Oct. 2020, leg. P. Czachura (KRAM F-60022; culture: P0020); • Świętokrzyskie Province, Kielce County, the Świętokrzyski National Park, Chełmowa Góra, on resin of Larixdeciduassp.polonica, 19 June 2021, leg. P. Czachura (KRAM F-60023; culture: P0021).

##### Notes.

The genus *Resinomelania* forms a distinct phylogenetic lineage close to the genera *Neocatenulostroma* and *Sthughesia*. *Resinomelania* differs from *Neocatenulostroma* by having one-septate or aseptate arthroconidia formed by disarticulation of hyphae. Moreover, *Resinomelania* frequently forms hyphal swellings and chlamydospores. In contrast, members of the genus *Neocatenulostroma* produce variously shaped, multiseptate conidia (= arthroconidia) with transverse, longitudinal, or oblique septa that arise from conidiophores, which may be aggregated into sporodochia or are formed by disarticulation of hyphae. Additionally, conidia in *Neocatenulostroma* are characterized by having rounded, pale cells at both sides (apex and base), while this feature is absent in *Resinomelania* (Butin et al. 1996; Ruibal et al. 2005; Crous et al. 2007a; Quaedvlieg et al. 2014). The morphological comparison of *Resinomelania* with *Sthughesia* is not possible since *Sthughesiajuniperi* is described from sexual morph (ascomata with asci and ascospores), although with information that hyphae are moniliform (Barr 1987a). Additionally, phialides were observed in this fungus (Barr 1987b). The morphology of *Resinomelaniacommunis*, with hyphae that develop into arthroconidia and chlamydospores, resembles the morphology of different genera and species of so-called “black yeasts”, which are phylogenetically distinct (Isola et al. 2016; Humphries et al. 2017; Trovão et al. 2019; Muggia et al. 2021; Piątek et al. 2024).

## ﻿Discussion

In this study, 13 strains of a dark, slow-growing fungus belonging to the class Dothideomycetes were isolated from resin outpourings of four conifer species (*Abiesalba*, Larixdeciduassp.polonica, *Piceaabies*, and *Pinussylvestris*) in Poland. Based on multi-locus sequence analyses, the strains were identified and described as a new species, *Resinomelaniacommunis*, accommodated in the newly established genus *Resinomelania*, which resides in the family Teratosphaeriaceae within the order Mycosphaerellales. Teratosphaeriaceae is the second-largest family within Mycosphaerellales, following the most speciose family, Mycosphaerellaceae (Hyde et al. 2024; Pem et al. 2024). Members of Teratosphaeriaceae exhibit diverse lifestyles; they are known as plant pathogens, saprobes, human pathogens, or animal inhabitants (Quaedvlieg et al. 2014; Duarte et al. 2017). Some species also thrive in extreme environments, including rocks, honeydew deposits, acidic or saline soils and waters, or near alcohol distilleries (Hujslová et al. 2013; Egidi et al. 2014; Scott et al. 2016; Czachura et al. 2021; Kolařík et al. 2021; Crous et al. 2023). *Resinomelaniacommunis*, as a fungus inhabiting resin outpourings of conifers—substrates rich in terpenoids and characterized by low water availability—may undoubtedly be considered an extremophilic species. It is, however, the first resinicolous fungus reported in both the family Teratosphaeriaceae and the order Mycosphaerellales. In this context, it is worth noting that another member of this family, *Queenslandipenidiellakurandae* (syn. *Penidiellakurandae*), was described from exudates of unidentified trees in Australia caused by fungal infection (Crous et al. 2007b; Quaedvlieg et al. 2014). However, it remains unclear whether these exudates were resin outpourings; presumably not, though further investigation is needed. Phylogenetically, *Resinomelaniacommunis* is most closely related to members of the genera *Neocatenulostroma* and *Sthughesia*.

The genus *Neocatenulostroma* comprises six species names, including one based on a validly described species (*Aulographinapinorum*), although its combination into *Neocatenulostroma* as *N.pinorum* is invalid [Art. F.5.1, ICNafp (Shenzhen)] (Butin et al. 1996; Taylor and Crous 2000; Crous et al. 2007a; Quaedvlieg et al. 2014; Phukhamsakda et al. 2022; Sastoque et al. 2024). It should be noted that *Aulographinapinorum* is the type species of the genus *Aulographina*, an older name that would have priority over the younger name *Neocatenulostroma*. The strains CBS 174.90 and CBS 302.71 of *Aulographinapinorum* used in previous phylogenetic analyses (Crous et al. 2009; Schoch et al. 2009; Sastoque et al. 2024) do not originate from the type specimen, and Cheewangkoon et al. (2012) considered it unlikely that they are authentic representatives of that species. Therefore, the taxonomic status of *Aulographinapinorum*/*Neocatenulostromapinorum* and the genera *Aulographina*/*Neocatenulostroma* requires further investigation using new cultures clearly linked to *A.pinorum*. Based on the phylogenetic analyses conducted in this study, only four species belong to *Neocatenulostroma* s. str. (Fig. 1). *Neocatenulostromacastaneae* clusters outside the strongly supported clade comprising the genera *Resinomelania*, *Neocatenulostroma* s. str., and *Sthughesia*. This placement is consistent with the analyses conducted by Phukhamsakda et al. (2022), where *N.castaneae* was described and grouped—albeit with weak support—on a long branch adjacent to *Neocatenulostroma* species. However, *Sthughesiajuniperi* was not included in their analyses. *Neocatenulostromacastaneae* should be accommodated in a distinct genus within the family Teratosphaeriaceae, a conclusion supported by its phylogenetic position and certain morphological features observed in culture, particularly the production of appressorial pegs, chlamydospores, and chlamydospore-like structures—traits not reported in species of *Neocatenulostroma* s. str. (Butin et al. 1996; Taylor and Crous 2000; Crous et al. 2007a; Quaedvlieg et al. 2014; Markovskaja et al. 2016; Phukhamsakda et al. 2022; Sastoque et al. 2024).

The genus *Sthughesia* includes only one species, *S.juniperi*, previously known as *Dimeriumjuniperi* or *Xenomerisjuniperi* (Dearness 1926; Müller and von Arx 1962; Barr 1987a, 1987b). It was originally described based on morphological characters (Barr 1987a) but later confirmed by molecular data (Winton et al. 2007). However, due to limited sampling of sequences within Teratosphaeriaceae, *S.juniperi* grouped closely with *Xenomerisraetica* in the study by Winton et al. (2007), as well as in the study conducted by Zhang et al. (2011). In the present study, sequences from all available strains of *Sthughesia* and *Xenomeris* spp. were analyzed in a broader phylogenetic context that included all genera in the family Teratosphaeriaceae (Fig. 1). This was necessary because our initial megablast query indicated that *Sthughesiajuniperi* (syn. *Xenomerisjuniperi*) was one of the closest relatives of *Resinomelaniacommunis*. The phylogenetic analyses clearly separated *S.juniperi* from the remaining *Xenomeris* species (Fig. 1). *Sthughesia* represents an independent generic lineage closely related to *Neocatenulostroma* s. str. and *R.communis*. This study reveals its phylogenetic placement for the first time.

A closer look at the ecology of the analyzed clade containing the genera *Neocatenulostroma* s. str., *Resinomelania*, and *Sthughesia* reveals several adaptations. Members of *Neocatenulostroma* have been reported as epiphytes or pathogens on living needles of *Abiesalba* and *Pinus* spp. (Butin et al. 1996; Markovskaja et al. 2016; Lazarević and Menkis 2020), on living leaves of *Proteacynaroides* (Taylor and Crous 2000; Quaedvlieg et al. 2014), on stones (Ruibal et al. 2005; Crous et al. 2007a; Quaedvlieg et al. 2014), on painted outdoor walls and PVC pipes of industrial warehouses (Quaedvlieg et al. 2014; Sastoque et al. 2024), as well as on skin lesions of human patients (Butin et al. 1996). *Sthughesiajuniperi* is a plant-associated species, isolated from a living branch of *Juniperusoccidentalis* (Dearness 1926). *Resinomelaniacommunis* is also a plant-associated fungus, but its resinicolous lifestyle distinguishes it from closely related genera and species. Its common occurrence on the resins of four different conifer species in the family Pinaceae indicates that it is a generalist in terms of host preference but a resinicolous species in terms of ecological strategy. Interestingly, only two ITS sequences available in GenBank were similar to those of *R.communis*. The first sequence, obtained from the bark beetle *Hylesinuscrenatus* on *Fraxinusexcelsior* in the Czech Republic (LR961841; named as *Neocatenulostromamicrosporum*; identities: 450/451, no gaps; M. Kolařík, unpubl.), differs from *R.communis* at only one nucleotide position, suggesting conspecificity. The second sequence (MH349092; named as *Neocatenulostromamicrosporum*; identities: 493/510, including 5 gaps), obtained from a lichen thallus on quartz in the extremely arid Atacama Desert in Peru (Díaz et al. 2023), is more divergent from *R.communis*, possibly representing a related but distinct species. However, the sequence is nearly identical along its entire length, with differences occurring only at the end of the read. Nevertheless, the widespread occurrence of *R.communis* on conifer resins and the rarity of related environmental sequences in GenBank from other substrates suggest that *R.communis* may exhibit a strong preference for resin outpourings.

In summary, this study provides new insights into the diversity of resinicolous fungi and the ecology of the order Mycosphaerellales, particularly the family Teratosphaeriaceae, by introducing the first genus and species exhibiting a resinicolous lifestyle in this fungal lineage. Plant resins represent a unique and chemically complex microhabitat in the natural environment. Fungi inhabiting such extreme and atypical environments often exhibit specialized metabolic pathways and adaptations. Therefore, microorganisms from these habitats hold high potential for numerous biotechnological applications (Tiquia-Arashiro and Grube 2019; Sahay 2022). Continued research on resinicolous fungi will contribute to a better understanding of this intriguing group within the fungal kingdom.

## Supplementary Material

XML Treatment for Resinomelania

XML Treatment for Resinomelania communis

## ﻿Additional information

### Use of AI

No use of AI was reported.

### Author ORCIDs

Paweł Czachura https://orcid.org/0000-0002-3562-8776

Marcin Piątek https://orcid.org/0000-0002-4968-2861

### Data availability

All data supporting the findings of this study are available in the GenBank nucleotide database (https://www.ncbi.nlm.nih.gov/genbank/), in the culture collections, and in the fungal herbarium, as referenced in Table 1 and the main text.
